# Protein N-glycosylation in oral cancer: Dysregulated cellular networks among *DPAGT1*, E-cadherin adhesion and canonical Wnt signaling

**DOI:** 10.1093/glycob/cwu031

**Published:** 2014-04-17

**Authors:** Xaralabos Varelas, Meghan P Bouchie, Maria A Kukuruzinska

**Affiliations:** 2 Department of Biochemistry, Boston University School of Medicine, Boston, MA, USA; 3 Department of Molecular and Cell Biology, Boston University School of Dental Medicine, Boston, MA, USA

**Keywords:** cancer, DPAGT1, E-cadherin, N-glycosylation, Wnt signaling

## Abstract

N-Linked glycosylation (N-glycosylation) of proteins has long been associated with oncogenesis, but not until recently have the molecular mechanisms underlying this relationship begun to be unraveled. Here, we review studies describing how dysregulation of the N-glycosylation-regulating gene, *DPAGT1*, drives oral cancer. *DPAGT1* encodes the first and rate-limiting enzyme in the assembly of the lipid-linked oligosaccharide precursor in the endoplasmic reticulum and thus mediates N-glycosylation of many cancer-related proteins. *DPAGT1* controls N-glycosylation of E-cadherin, the major epithelial cell–cell adhesion receptor and a tumor suppressor, thereby affecting intercellular adhesion and cytoskeletal dynamics. *DPAGT1* also regulates and is regulated by Wnt/β-catenin signaling, impacting the balance between proliferation and adhesion in homeostatic tissues. Thus, aberrant induction of *DPAGT1* promotes a positive feedback network with Wnt/β-catenin that represses E-cadherin-based adhesion and drives tumorigenic phenotypes. Further, modification of receptor tyrosine kinases (RTKs) with *N*-glycans is known to control their surface presentation via the galectin lattice, and thus increased *DPAGT1* expression likely contributes to abnormal activation of RTKs in oral cancer. Collectively, these studies suggest that dysregulation of the *DPAGT1*/Wnt/E-cadherin network underlies the etiology and pathogenesis of oral cancer.

## Introduction

Protein N-glycosylation is one of the most abundant posttranslational modifications in biology (Aebi 2013). Through its effects on protein folding, targeting, clearance, secretion and conformation, N-glycosylation controls a broad spectrum of cellular functions that are vital for development and homeostasis, including cell–cell and cell–matrix adhesion, cell proliferation, cell survival and immune system responses (Stanley et al. 2009; Varki and Lowe 2009). Despite the importance of N-glycosylation in various cellular processes, the molecular mechanisms by which N-glycosylation directs signaling networks or how the genes that encode enzymes in the N-glycosylation pathway are regulated are only starting to be unraveled.

Dysregulated N-glycosylation is a common theme in disease, including a prevalent association with cancer (Varki and Freeze 2009; Varki et al. 2009). Cancer develops through a multistep process that includes mechanisms that promote tumor initiation, and progression, accompanied by complex pathology characterized by the accumulation of epigenetic, genetic and cytogenetic changes (Hanahan and Weinberg 2011; Valastyan and Weinberg 2011). In tumors of epithelial origin (carcinomas), cancer progression is associated with dramatic changes in cell–cell or E-cadherin-mediated adhesion, as well as aberrant organization of cell polarity and cytoskeleton architecture (Beavon 2000; Conacci-Sorrell et al. 2002; Wodarz and Nathke 2007). Dysregulated signaling pathways contribute to the ability of tumor cells to proliferate, evade cell death and, in extreme cases, to metastasize into distant tissues (Dhillon et al. 2007). Studies have emerged describing N-glycosylation as a key regulator of various aspects of tumorigenesis, thus indicating that this posttranslational modification is a major player in the early development and progression of cancer (Lau and Dennis 2008; Guo et al. 2010).

In this review, we highlight the emerging roles of N-glycosylation in cancer with a particular focus on oral squamous cell carcinoma (OSCC). OSCC involves epithelial neoplasms of the oral cavity and oropharynx, and it ranks as one of the most morbid cancers whose incidence is on the rise (Choi and Myers 2008; Leemans et al. 2011; Rothenberg and Ellisen 2012). We discuss evidence for how N-glycosylation impacts cell adhesion and cytoskeletal dynamics, as well as how the N-glycosylation pathway directs key oncogenic signaling pathways, such as the Wnt/β-catenin pathway (also known as the canonical Wnt pathway).

## *DPAGT1* as a key regulator of protein N-glycosylation in homeostasis and oral cancer

**Fig. 1.**
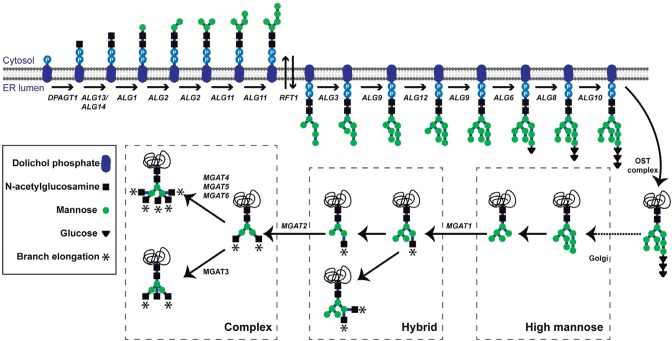
Simplified scheme of the N-glycosylation pathway. N-Glycosylation is initiated in the endoplasmic reticulum (ER) by the GPT, encoded by the *DPAGT1* gene. GPT catalyzes the transfer of GlcNAc from UDP-GlcNAc to dolichol-phosphate to produce dolichol-PP-GlcNAc, which is the first step in the synthesis of LLO precursor. Subsequently, LLO is transferred co-translationally to newly synthesized polypeptides. After initial processing steps in the ER, glycoproteins transit to the Golgi, where *N*-glycans are further modified, giving rise to mature N-glycoproteins modified with oligosaccharides ranging from high mannose/hybrid to complex.

N-Glycosylation is initiated in the endoplasmic reticulum (ER) by the dolichol phosphate-dependent *N*-acetylglucosamine 1-phospho-transferase (GPT), encoded by the *DPAGT1* gene (Rine et al. 1983; Bretthauer 2009; Aebi 2013). GPT catalyzes the transfer of *N*-acetylglucosamine (GlcNAc) from UDP-GlcNAc to dolichol phosphate to produce dolichol-PP-GlcNAc, which is the first step in the synthesis of a lipid-linked oligosaccharide (LLO) precursor (Figure 1). Subsequently, LLO is transferred co-translationally to newly synthesized polypeptides (Helenius and Aebi 2001; Aebi 2013). After initial processing steps in the ER, glycoproteins transit to the Golgi, where *N*-glycans are further modified, giving rise to mature N-glycosylated proteins (*N*-glycoproteins) decorated with oligosaccharides ranging from high mannose/hybrid to complex (Helenius and Aebi 2001; Rini et al. 2009) (Figure 1).

### Functional significance

The N-glycosylation pathway is conserved across eukaryotes, and the essential nature of proper *N*-glycan regulation is highlighted from work in model organisms. For example, deletion of the *Saccharomyces cerevisiae* ortholog of *DPAGT1*, *ALG7*, is lethal (Kukuruzinska and Robbins 1987) and the presence of a hypomorphic *alg7* allele is associated with dysregulation of metabolic and signaling pathways, including those involved in cellular differentiation and cell wall signaling (Klebl et al. 2001; Mendelsohn et al. 2005). Deletion of *DPAGT1* in mice leads to peri-implantation mortality (Marek et al. 1999), documenting its essential role at the earliest stages of mammalian development. In humans, mutations in *DPAGT1* result in a significant reduction in GPT activity, giving rise to congenital disorders of glycosylation (CDG-Ij) and early mortality (Wu et al. 2003; Carrera et al. 2012; Timal et al. 2012). Tunicamycin, an analog of UDP-GlcNAc and antibiotic inhibitor of GPT, causes cell death in all cellular systems examined to date (Tkacz and Lampen 1975; Lehle and Tanner 1976; Heifetz et al. 1979). While depletion of *DPAGT1* results in severe disorders, including hypotonia, mental retardation and hypokinesia (Carrera et al. 2012), its overexpression is linked to oral tumorigenesis (Nita-Lazar et al. 2009). In cultured epithelial cells, overexpression of *DPAGT1* drives cell proliferation, changes in cell morphology and gene expression resembling an epithelial-to-mesenchymal transition (EMT) (Sengupta et al. 2013). Studies indicate that *DPAGT1* regulates *N*-glycan site occupancy, where a decrease in its expression is likely to result in glycoprotein misfolding, while its overexpression may promote the utilization of unused *N*-glycan addition sites to generate novel glycoforms (Mendelsohn et al. 2005; Liu et al. 2013).

*DPAGT1* functions at a rate-limiting step in the N-glycosylation pathway, so modest changes in its expression result in robust changes in the N-glycosylation status of proteins (Clark et al. 1983; Hayes and Lucas 1983; Welply et al. 1985; Meissner et al. 1999; Mendelsohn et al. 2005; Bretthauer 2009). Human fibroblasts from a patient bearing mutations in both alleles of *DPAGT1* display dramatically reduced GPT activity that is associated with diminished levels of LLO and hypo-glycosylation of proteins (Wu et al. 2003). Similarly, a hypomorphic allele of *alg7* in budding yeast that produces 50% of GPT has a 6-fold reduction in LLO levels and severe hypo-glycosylation of proteins (Mendelsohn et al. 2005). Downregulation of LLO levels in yeast, in turn, leads to the rewiring of signaling networks and altered cell adhesion and cell wall sensing (Klebl et al. 2001). Furthermore, cells bearing the *alg7* hypomorphic allele display a “clumping” phenotype, suggesting altered cell surface properties and increased adhesion (Mendelsohn et al. 2005).

### Regulation of DPAGT1 expression

The human *DPAGT1* gene maps to chromosome 11q23 (Regis et al. 2002). Both yeast *ALG7* and mammalian *DPAGT1* exhibit transcript complexity (Kukuruzinska and Robbins 1987; Lehrman et al. 1988; Huang et al. 1998). The yeast *ALG7* gene produces two major transcripts, 1.4 and 1.6 kb, which differ in the lengths of their 3′UTRs (Kukuruzinska and Robbins 1987). These 3′UTR differences are biologically significant, as the 1.4 kb transcript is less stable but more translationally competent than the 1.6 kb species (Lennon et al. 1997). Mapping of rodent *DPAGT1* mRNAs also revealed multiple transcripts, including 1.9 and 2.2 kb, that exhibit identical 5′ ends but different 3′UTR lengths (Huang et al. 1998). These transcripts are predicted to give rise to GPT of 408 amino acids with a molecular weight of 46 kDa. Interestingly, a third transcript has been shown to map downstream from the 5′ end of the 1.9 and 2.2 kb mRNAs (Huang et al. 1998); the 1.5 kb transcript is predicted to give rise to a GPT isoform lacking the first 107 N-terminal amino acids and with a molecular weight of 35 kDa. These transcripts produce biologically different GPT isoforms that contain either one or two dolichol recognition domains (DRDs), with the first dolichol-binding site mapping to the most N-terminal 100 amino acids that is absent from the 1.5 kb transcript. Although earlier studies suggested that both DRDs were required for GPT function (Datta and Lehrman 1993), transfection of a *DPAGT1* cDNA encoding the short 1.5 kb mRNA variant produces phenotypes similar to the cDNA encoding the full-length transcript (Sengupta et al. 2013). Further studies are needed to decipher the precise roles of mammalian *DPAGT1* transcript heterogeneity.

In many cellular systems, changes in GPT activity reflect transcriptional regulation of *DPAGT1*. In yeast, *ALG7* transcripts are modulated with growth and differentiation, and changes in their abundance have profound effects on the N-glycosylation status of proteins (Kukuruzinska and Lennon 1995; Pretel et al. 1995; Kukuruzinska and Lennon-Hopkins 1999; Mendelsohn et al. 2005). Withdrawal of glucose from exponentially growing yeast cells leads to dramatic reduction of *ALG7* mRNA, whereas addition of glucose to growth arrested cells results in a robust increase in *ALG7* transcript levels in the presence of cycloheximide, suggesting transcriptional derepression (Kukuruzinska and Lennon 1994). *DPAGT1* expression is also regulated during mouse mammary gland development, as well as with hormonal insulin, glucocorticoid receptor or prolactin stimulation (Rajput et al. 1994; Ma et al. 1996). In mouse P19 teratocarcinoma cells, *DPAGT1* transcription is regulated by retinoic acid (Meissner et al. 1999). Hamster, canine and human *DPAGT1* expression is also upregulated with mitogenic signals and with canonical Wnt signaling (Sengupta et al. 2010). Consistent with these regulatory cues, transcriptional expression of *DPAGT1* is inappropriately induced in oral carcinoma (Nita-Lazar et al. 2009; Jamal et al. 2012). Conversely, reduced expression of *DPAGT1* is associated with increased cell adhesion and cellular differentiation (described under *DPAGT1 regulates E-cadherin N-glycosylation status and AJ maturity*) (Fernandes et al. 1999; Nita-Lazar et al. 2009, 2010).

**Fig. 2.**
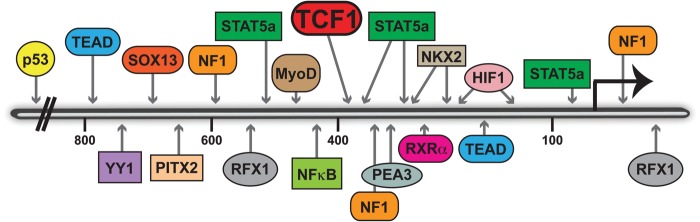
The organization of the *DPAGT1* promoter. The promoter of *DPAGT1* has potential binding sites for numerous transcription factors, as illustrated. These factors affect diverse cellular functions, providing a platform for integrating N-glycosylation into the complex networks of signaling and metabolic pathways, including those involved in cell adhesion, proliferation, survival, hypoxia and stress response.

The promoter of *DPAGT1* has potential binding sites for a plethora of transcription factors, and it is estimated that close to 200 transcription factors bind to the *DPAGT1* promoter. Some of these transcription factors have acknowledged roles in important cellular processes and cancer, including TCF, STAT, p53, TEAD, HIF1, Nkx2, MyoD, SOX13 and RXRα (Figure 2). These factors affect expression of genes involved in diverse cellular functions and thus provide a platform for integrating N-glycosylation into the complex networks of signaling and metabolic pathways and structural processes, including those involved in cellular proliferation, survival, hypoxia, stress response and T-cell lineage and immunity. To date, STAT5a has been shown to bind to the mouse mammary *DPAGT1* promoter in vitro and to stimulate *DPAGT1* transcription in COS7 cells (Zhang et al. 2003). Moreover, TCF proteins, which function in the canonical Wnt signaling pathway, bind to the *DPAGT1* promoter in vivo (Sengupta et al. 2010). Canonical Wnt effectors, β- and γ-catenins, are recruited to TCF transcription factors at the *DPAGT1* promoter to activate its transcription (Sengupta et al. 2010; Jamal et al. 2012). Aberrant activation of canonical Wnt signaling in OSCC is associated with increased binding of β- and γ-catenins to TCF in the *DPAGT1* promoter and elevated expression of *DPAGT1* (Jamal et al. 2012). Human specimens of OSCC display induced expression of *DPAGT1* and GPT protein levels*,* which is associated with increased modification of E-cadherin and collagen triple helix repeat containing 1 (CTHRC1) with complex *N*-glycans (Nita-Lazar et al. 2009; Liu et al. 2013). Consistent with a fundamental role in cancer, overexpression of *DPAGT1* occurs in cancer cell lines derived from a multitude of tumors from different oral sites (Nita-Lazar et al. 2009; Jamal et al. 2012). Furthermore, treatment of cell lines derived from tumors of different origins and of tumor mouse models in vivo with tunicamycin, an inhibitor of GPT, has been shown to reduce tumor growth (Shiraishi et al. 2005; Hiss et al. 2007; de-Freitas-Junior et al. 2012; Hou et al. 2013). Thus, it is likely that altered *DPAGT1* expression is an important mechanism by which N-glycosylation is dysregulated in cancer.

### Coordinate regulation of N-glycosylation genes

In budding yeast, transcripts of N-glycosylation genes encoding glycosyltransferases downstream from *ALG7* in the LLO pathway are co-regulated with *ALG7*, suggesting coordinate gene regulation (Kukuruzinska and Lennon 1994; Lennon et al. 1995). In mammalian systems, in addition to regulating LLO abundance and the extent of protein N-glycosylation, *DPAGT1* affects the quality of *N*-glycan structures in the Golgi. Upregulation of *DPAGT1* mRNA is associated with increased expression of *ALG1*, a gene that encodes a component of the ER, as well as increased expression of *MGAT5*, a gene encoding a Golgi protein (Sengupta et al. 2013). Since Golgi N-glycosylation-regulating enzymes control the assembly of macromolecular complexes at the cell surface, it is likely that dynamic organization of the galectin lattice involves a synchronized regulation with *DPAGT1* expression (Lau et al. 2007; Dennis et al. 2009). In addition, *MGAT5* may be affected by signaling pathways regulated by DPAGT1 (see below). In Madin-Darby canine kidney (MDCK) cells, siRNA-mediated reduction of *DPAGT1* expression by only 40% results in a striking depletion of complex *N*-glycan modification of E-cadherin (Nita-Lazar et al. 2010). Conversely, a 2.5-fold increase in *DPAGT1* expression by induction with the canonical Wnt ligand, Wnt3a, leads to greatly enhanced modification of E-cadherin with complex *N*-glycans (Sengupta et al. 2010). Taken together, these data suggest that the Golgi-regulating genes respond synchronously with changes to *DPAGT1* expression, and that coordinate regulation of the N-glycosylation pathway is critical for determining complex N-glycosylation patterns observed upon changing developmental, physiological and environmental cues.

## *DPAGT1*/N-glycosylation as a regulator of intercellular adhesion and cytoskeletal dynamics

Cell–cell adhesion directs cytoskeletal dynamics to establish and maintain cellular polarity, which ultimately determines accurate tissue architecture (Mays et al. 1995; Nejsum and Nelson 2007; Desai et al. 2009). Dysregulated cell adhesion is a hallmark of many diseases, most notably cancer (Wei et al. 2002; Onder et al. 2008). Interestingly, modification of key adhesion and cytoskeletal-regulating proteins with *N*-glycans controls their activity, and thus dysregulated N-glycosylation may contribute to altered adhesive properties observed in disease (Guo et al. 2003, 2009, 2012; Liwosz et al. 2006; Vagin et al. 2008; Jamal et al. 2009; Nita-Lazar et al. 2010; Langer et al. 2012). Given the importance of this relationship, the links between the regulation of N-glycosylation and cell adhesion are starting to be explored. We summarize some recent findings in this section.

### DPAGT1 regulates E-cadherin N-glycosylation status and AJ maturity

**Fig. 3.**
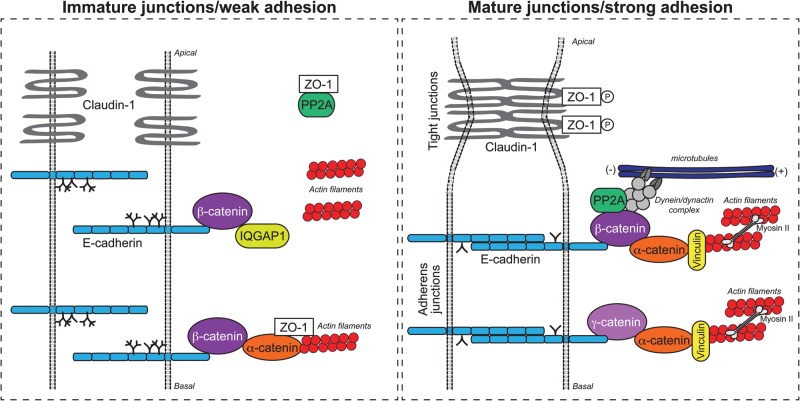
Schematic of how *N*-glycosylation of E-cadherin affects intercellular adhesion and cytoskeletal dynamics. Immature or weak AJs comprise N-glycosylated E-cadherin/β-catenin complexes which interact IQGAP1, thereby repressing the recruitment of α-catenin and local actin filaments. Conversely, mature AJs display hypo-glycosylated E-cadherin/β-catenin-complexes that interact with PP2A, thereby restricting its phosphatase activity away from ZO-1 and claudin-1. This allows ZO-1 and claudin-1 phosphorylation, leading to stabilization of TJs. Hypo-glycosylated V13/β-catenin complexes also recruit dynein/dynactin to tether microtubules. Additionally, hypo-glycosylated E-cadherin/γ-catenin complexes recruit α-catenin and vinculin, which promotes the interaction of AJs with the actin filaments.

The N-glycoprotein E-cadherin is the major adhesion receptor that mediates homotypic cell–cell interactions in epithelial cells (Takeichi 1991; Wheelock and Johnson 2003; Gumbiner 2005). E-cadherin is a single span transmembrane protein-containing five extracellular domains, or ectodomains (ECs), a transmembrane region and a cytoplasmic tail. The ECs dimerize in a Ca^2+^-dependent manner and interact with E-cadherin dimers on adjacent cells to form cell–cell contacts. The stability of E-cadherin contacts is regulated by the cytoplasmic domain of E-cadherin, which organizes the recruitment of multiprotein complexes that make up the adherens junctions (AJs) to mediate associations with the cellular cytoskeleton (Jamora and Fuchs 2002; Hartsock and Nelson 2008). Primordial, or nascent, AJs consist of the core AJ components, E-cadherin/β-catenin/α-catenin, which are frequently associated with ZO-1, a scaffold protein that organizes tight junctions (TJs) apically from AJs (Rajasekaran et al. 1996; Ando-Akatsuka et al. 1999). This sequestration of ZO-1 by immature AJs is thought to prevent TJ formation when AJs are still unstable. In some immature junctions, E-cadherin/β-catenin is directly associated with IQGAP1, a known junction destabilizer that competes with α-catenin for the binding to β-catenin (Kuroda et al. 1998). Nascent AJs do not exhibit extensive interactions with the actin cytoskeleton or microtubules (Ligon et al. 2001; Stehbens et al. 2006; Nejsum and Nelson 2007; Hong et al. 2013). In contrast, large multiprotein scaffolds define mature AJs by recruiting γ-catenin and α-catenin as well as actin-binding and actin-cross-linking proteins, such as vinculin and α-actinin (Rudiger 1998; Watabe-Hchida et al. 1998; Imamura et al. 1999; Vasioukhin et al. 2001; Nemade et al. 2004; Drees et al. 2005; Maiden and Hardin 2011; Twiss et al. 2012). Protein phosphatase 2A (PP2A) also has a key role in organizing mature AJs since PP2A dephosphorylates ZO-1 and other components of TJs to interfere with their assembly into scaffolds and to form functional TJs (Sontag 2001; Tar et al. 2004). When mature AJs form, they sequester PP2A and lose association with ZO-1, thus enabling ZO-1 to become phosphorylated and to move apically and assemble functional TJs (Jain et al. 2011) (Figure 3).

EC4 and EC5 of human E-cadherin have four *N*-glycan addition sites that, depending on the physiological state, are modified with either complex or hybrid/high mannose oligosaccharides (Liwosz et al. 2006; Nita-Lazar et al. 2009). Sites 1 on EC4 and 3 on EC5 are preferentially modified with *N*-glycans, while sites 2 and 4 on EC4 and EC5, respectively, have either no *N*-glycans or unsubstantial structures (Liwosz et al. 2006) (Sengupta and Kukuruzinska, unpublished). The N-glycosylation status of E-cadherin is dynamic and subject to cell density changes (Liwosz et al. 2006). In sparse cells, E-cadherin exhibits a higher molecular size than in dense cells, and this size difference can be eliminated by treatment with PNGaseF, an amidase that removes most *N*-glycans from *N*-glycoproteins. This higher molecular size of E-cadherin in sparse cells is due to large complex *N*-glycans at site 1 on EC4, and high mannose/hybrid *N*-glycans at site 3. Accordingly, E-cadherin from sparse cells is mostly resistant to treatment with EndoH, an endoglycosidase that removes high mannose/hybrid *N*-glycans. In contrast, dense cultures typically produce E-cadherin with high mannose/hybrid structures, suggesting loss of complex *N*-glycans from site 1 (Liwosz et al. 2006) (Sengupta and Kukuruzinska, unpublished). These density-dependent changes in the E-cadherin N-glycosylation status are significant, since sparse cells are known to form primordial, or immature, AJs while dense cultures form mature adhesion belts that drive stable cell–cell contacts. Thus, extensive N-glycosylation of E-cadherin with complex *N*-glycans is associated with nascent AJ while hypo-glycosylation of E-cadherin reflects their mature status. Interestingly, hypo-glycosylation does not appear to impact the trafficking of E-cadherin to the cell surface (Nita-Lazar et al. 2010). Nonetheless, hypo-glycosylated E-cadherin is associated with diminished abundance, suggesting that N-glycosylation impacts its stability (Liwosz et al. 2006; Sengupta et al. 2013). Although the mechanisms underlying the effects of N-glycosylation on E-cadherin junctional maturity remain unclear, recent studies with the neural cadherin (N-cadherin) suggest that *N*-glycans do not affect the initial interactions between cadherin molecules *in trans*, but that subsequent additional cadherin interactions are controlled by N-glycosylation (Langer et al. 2012).

Several lines of evidence indicate that *DPAGT1* regulates the N-glycosylation status of E-cadherin. Partial knockdown of *DPAGT1* expression with siRNA results in the production of hypo-glycosylated E-cadherin, which organizes mature AJs and exhibits increased interaction with the actin cytoskeleton and microtubules (Jamal et al. 2009; Nita-Lazar et al. 2010). Furthermore, knockdown of *DPAGT1* expression in A253 and CAL27 head and neck cancer cells reverts their mesenchymal-like phenotype to an epithelial morphology by remodeling AJs to promote interaction with γ- and α-catenins and vinculin (Nita-Lazar et al. 2009; Jamal et al. 2012). Conversely, induced *DPAGT1* expression in MDCK cells leads to increased modification of E-cadherin with complex *N*-glycans and its reduced membrane association (Sengupta et al. 2010, 2013) (Figure 3).

Studies using a hypo-glycosylated mutant of E-cadherin, V13, generated by deleting two major complex and high mannose/hybrid *N*-glycan addition sites, further confirm the enhanced ability for hypo-glycosylated E-cadherin to form mature AJs. These effects are observed in Chinese Hamster Ovary (CHO) cells that lack endogenous E-cadherin (Liwosz et al. 2006), with similar results observed in MDCK cells, which is consistent with V13 E-cadherin acting as a dominant active mutation (Liwosz et al. 2006; Nita-Lazar et al. 2010). Several possible models for how reduced N-glycosylation of E-cadherin promotes molecular remodeling of AJs can be proposed from the reported data and include: (i) hypo-glycosylated E-cadherin associates with γ-catenin more efficiently than with β-catenin; (ii) hypo-glycosylated E-cadherin/γ-catenin complexes preferentially interact with α-catenin and vinculin; (iii) hypo-glycosylated E-cadherin distributes with Triton-insoluble membrane fraction; (iv) hypo-glycosylated E-cadherin complexes interact more with PP2A; and (v) hypo-glycosylated E-cadherin junctions exhibit reduced interaction with ZO-1 and IQGAP1 (Figure 3). The mechanism by which N-glycosylation affects the molecular organization of E-cadherin protein complexes on the cytoplasmic side remains unclear, although it is likely to involve a conformational change in *cis* that leads to enhanced interaction with junction stabilizing and actin-binding/cross-linking proteins.

Many epithelial cancers display loss of E-cadherin, and E-cadherin is commonly downregulated in tumors by transcriptional repressors such as SLUG, SNAIL, TWIST and ZEB (Schipper et al. 1991; Doki et al. 1993; Gofuku et al. 1999; Wei et al. 2002; Bolos et al. 2003; Conacci-Sorrell et al. 2003; Wang et al. 2011; Wu et al. 2012). However, in some cancers, notably a major subset of OSCCs, E-cadherin is not lost. Rather, due to overexpression of *DPAGT1*, E-cadherin is highly modified with complex *N*-glycans and unable to form mature cell–cell contacts (Nita-Lazar et al. 2009). Notably, OSCC AJs exhibit almost complete loss of α-catenin, vinculin and PP2A and exhibit increased interaction with IQGAP1, a known junction destabilizer (Nita-Lazar et al. 2009). This suggests that in OSCC, and most likely in other epithelial tumors that maintain E-cadherin expression, N-glycosylation is a key determinant of reduced E-cadherin adhesion. Indeed, E-cadherin in breast tumors exhibits highly branched *N*-glycans on extracellular domains EC4 and EC5, and hyper-glycosylation at these sites destabilizes epithelial junctions and increases tumor progression (Pinho et al. 2009).

### DPAGT1 impacts the assembly of TJs

Examination of the molecular organization of TJs in cells with partially downregulated *DPAGT1* expression and cells bearing the hypo-glycosylated E-cadherin variant reveals that hypo-glycosylated E-cadherin promotes the assembly of robust TJs. This is reflected by an increased abundance of phosphorylated ZO-1 and claudin-1 and by a greater molar ratio of claudin-1 bound to ZO-1 protein complexes. Moreover, these molecular changes in TJs are physiologically significant as they result in a higher transepithelial resistance (Nita-Lazar et al. 2010). Thus, by affecting the composition and maturity of AJs, N-glycosylation indirectly controls the assembly of TJs (Figure 3). Given the key roles of TJs in cell density sensing and the establishment of apical–basal polarity, N-glycosylation is also a likely contributor to these processes. The high N-glycosylation levels found in OSCC may therefore drive the loss of TJs and epithelial polarity, accounting for the mislocalization of ZO-1 from the junctional regions to the cytoplasm in oral cancer (Nita-Lazar et al. 2009).

### DPAGT1/N-glycosylation as a regulator of cytoskeletal dynamics

N-Glycosylation-dependent differences observed in the composition of AJs are thought to impact AJ interactions with the cytoskeleton. Immunofluorescence imaging and double immunoprecipitation experiments have shown that hypo-glycosylated E-cadherin/β-catenin complexes preferentially interact with PP2A and dynein/dynactin that, in turn, associate with microtubules (Jamal et al. 2009). In contrast, E-cadherin/γ-catenin complexes interact better with α-catenin and vinculin, indicating their association with the actin cytoskeleton (Liwosz et al. 2006; Jamal et al. 2009; Nita-Lazar et al. 2010). It is possible that PP2A collaborates with α-catenin and vinculin in promoting the association of hypo-glycosylated E-cadherin complexes with the cytoskeleton by mediating their interaction with microtubules. PP2A dephosphorylates the Tau protein, which is one of the microtubule-associated proteins, and dephosphorylated Tau promotes microtubule assembly. Furthermore, PP2A impacts phosphorylation of EB1, the microtubule plus-end-tracking protein, which may affect association of p150 (Glued) with the dynein/dynactin complex and consequently tethering to AJs. PP2A may therefore affect the transport of polarity protein-containing vesicles from the trans-Golgi network to AJs and help establish apical–basal epithelial polarity. Thus, the N-glycosylation status of E-cadherin has a central role in organizing the composition and cytoskeletal association of E-cadherin AJs and TJs (Figure 3).

Increased association of hypo-glycosylated E-cadherin/γ-catenin complexes with α-catenin is likely to affect the local concentration of α-catenin and to promote α-catenin dimerization concomitant with the inhibition of Arp2/3 complexes, leading to decreased actin branching and increased actin bundling and cable formation (Hartsock and Nelson 2008; Maiden and Hardin 2011). Also, greater interaction of hypo-glycosylated E-cadherin complexes with vinculin would promote actin bundling and enhance local α-catenin function and interaction with the actin cytoskeleton (Twiss et al. 2012). This scenario is consistent with preferential association of hypo-glycosylated E-cadherin with the Triton-insoluble membrane fraction (Liwosz et al. 2006). The recruitment of vinculin to hypo-glycosylated E-cadherin junctions occurs in a myosin II-dependent manner, augmenting AJ mechanosensing (le Duc et al. 2010). In addition, interactions between E-cadherin and the actin cytoskeleton are critical for limiting E-cadherin diffusion within the membrane and for the increased packing of junctions and eventually for their apical migration (Hong et al. 2013). The E-cadherin/F-actin interface plays critical roles in transducing physical forces at cell–cell contacts into cellular signaling (Huveneers and de Rooij 2013). Thus, by regulating the composition of E-cadherin junctions and their interaction with the actomyosin cytoskeleton, N-glycosylation indirectly regulates their mechanosensory capacity and mechanotransduction at cell–cell junctions.

### Other N-glycosylation genes in E-cadherin-mediated adhesion

The Golgi N-glycosylation-regulating enzymes have also been reported to impact cell–cell adhesion and to play a role in cancer (Dennis et al. 1987; Kitada et al. 2001; Guo et al. 2003, 2009; Pinho et al. 2009, 2013). Upregulation of *MGAT5,* a glycosyltransferase that extends *N*-glycans into highly branched structures, promotes cell migration and metastases in vivo (Guo et al. 2003). The *N*-glycan products of *MGAT5* have been shown to interfere with cadherin adhesion and with the formation of tight adhesion belts (Guo et al. 2003; Vagin et al. 2008). Conversely, increased expression of *MGAT3*, which encodes a glycosyltransferase responsible for the addition of a bisecting GlcNAc to the chitobiose core of *N*-glycans, interferes with the formation of branched *N*-glycan structures and enhances cell–cell adhesion, thus functioning as a tumor suppressor (Kitada et al. 2001; Pinho et al. 2012). These collective findings indicate that increased N-glycosylation and the generation of complex, highly branched structures promote tumorigenesis by interfering with intercellular adhesion.

## *DPAGT1*/N-glycosylation in Wnt signaling

Cancers are typically associated with dysregulated signaling through pathways that normally have vital roles in embryonic development. The canonical Wnt signaling pathway is one such pathway which, when left unchecked, drives early pathogenesis and/or the metastasis of a range of cancers, including OSCC (Polakis 2000; MacDonald et al. 2009; Clevers and Nusse 2012). Activation of canonical Wnt signaling involves the binding of Wnt ligands to LRP/Frizzled receptor complexes, triggering intracellular signaling cascades that prevent the phosphorylation of the transcriptional regulator, β-catenin, and subsequently lead to stabilization of β-catenin protein levels. β-catenin thereby accumulates in the nucleus and interacts with the TCF/LEF family of transcription factors to control target gene expression. Over the past few years, data have emerged linking the canonical Wnt pathway with the N-glycosylation pathway, indicating that the network formed between these signals may be the underlying force driving tumorigenesis (Sengupta et al. 2010; Xu et al. 2011).

### DPAGT1 is a target of canonical Wnt signaling

Several pieces of evidence indicate that *DPAGT1* is a direct target of the canonical Wnt pathway. First, treatment of cultured cells with either a Wnt pathway activator, LiCl, or with its ligand, Wnt3a, results in increased *DPAGT1* expression in canine MDCK, hamster CHO, human A253 salivary epidermoid carcinoma and human CAL27 OSCC cells (Sengupta et al. 2010). Second, both β- and γ-catenins are recruited to the *DPAGT1* promoter at TCF/LEF sites in vitro and in vivo (Sengupta et al. 2010). Third, mapping of the *DPAGT1* promoter has revealed that the TCF/LEF binding region is sufficient to drive the expression of a luciferase reporter in response to Wnt. Interestingly, the control of canonical Wnt signaling by cell density may be an important regulator of *DPAGT1* expression, since reduced *DPAGT1* expression is associated with the diminished canonical Wnt activity observed in dense cells. High cell density conditions correlate with a decreased occupancy of the *DPAGT1* promoter by β- and γ-catenins determined by chromatin immunoprecipitation assays and with reduced Wnt-specific TOP-Flash luciferase reporter activity (Sengupta et al. 2013). These observations, therefore, indicate that *DPAGT1* provides an important point of crosstalk between the Wnt and N-glycosylation pathways (Sengupta et al. 2010).

Evidence suggests that the connections between *DPAGT1* and canonical Wnt signaling are important for cancer development and progression. In OSCC, overexpression of *DPAGT1* is linked to aberrant activation of canonical Wnt signaling (Jamal et al. 2012). OSCC tissues display great increases in total levels of β- and γ-catenins, and this correlates with their increased occupancy at the *DPAGT1* promoter. Aberrant activation of canonical Wnt signaling in OSCC is also associated with reduced expression of DKK-1, which is a negative feedback inhibitor of the Wnt pathways (Jamal et al. 2012). Under normal conditions, *DKK1* itself is a target of canonical Wnt signaling and its induction by β-catenin leads to the eventual inhibition of canonical Wnt signaling, including subsequent decreased expression of *DPAGT1* and other Wnt targets. Such built-in control assures that canonical Wnt is not activated for inappropriate lengths of time and the loss of DKK-1 in OSCC may be an important mechanism driving cancer progression. Similar inhibition of DKK-1 has been observed in colorectal cancer, which results from the epigenetic modification of the *DKK1* promoter (Aguilera et al. 2006). Although the mechanism underlying the loss of DKK-1 in OSCC is unknown, treatment of OSCC CAL27 cells with a methylation inhibitor, 5-aza-2′-deoxycytidine, results in the inhibition of *DPAGT1* expression, suggesting epigenetic repression of *DKK1* in oral cancer (Sengupta and Kukuruzinska, unpublished).

### DPAGT1 regulates canonical Wnt signaling

Activation of *DPAGT1* expression by Wnt signaling induces canonical Wnt activity by controlling N-glycosylation of its upstream regulatory components. N-Glycosylation is required for efficient membrane targeting of Wnt3a and LRP5/6 (Khan et al. 2007; Komekado et al. 2007; Jung et al. 2011). Sparse cells in which *DPAGT1* expression is knocked down with siRNA show decreased *DPAGT1* promoter activity and reduced Wnt activity. Moreover, silencing of *DPAGT1* expression leads to the retention and diminished membrane localization of hypo-glycosylated Wnt3a and LRP5/6 and reduced canonical Wnt signaling (Sengupta et al. 2013). These findings indicate that *DPAGT1* and canonical Wnt function in a positive feedback loop which, when activated, may drive disease states, including cancer (Jamal et al. 2012).

### Mature AJs inhibit DPAGT1 and canonical Wnt signaling: Feedback loops among DPAGT1, canonical Wnt and E-cadherin adhesion

In addition to its function as a transcriptional effector of the canonical Wnt pathway, β-catenin is a key structural component of AJs (Brembeck et al. 2006). Likewise, γ-catenin also functions as both a transcriptional effector of canonical Wnt signaling and as a junctional component (Zhurinsky et al. 2000; Maeda et al. 2004; Shimizu et al. 2008). Such sharing of β- and γ-catenins by canonical Wnt signaling, *DPAGT1* and E-cadherin adhesion provides a platform for the intimate coordination of their activities during development and in tissue homeostasis (Heuberger and Birchmeier 2010; Sengupta et al. 2013).

**Fig. 4.**
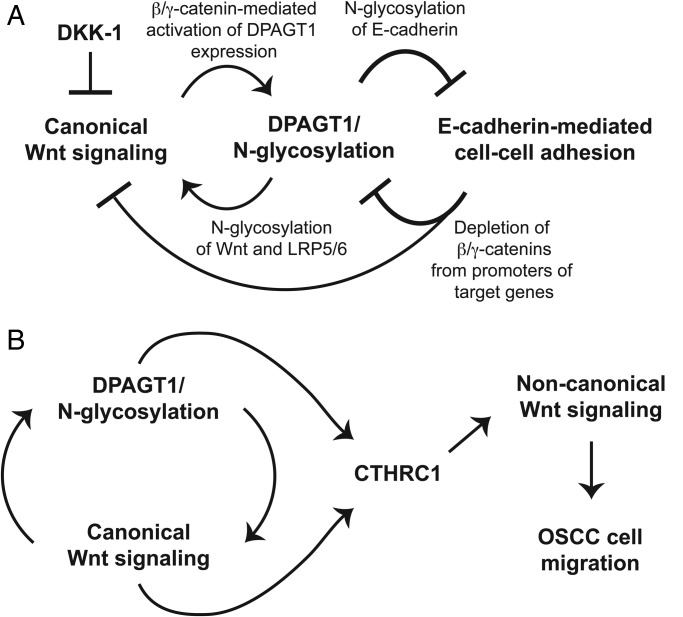
*DPAGT1*/Wnt/E-cadherin network. (**A**) Signaling network of *DPAGT1*, canonical Wnt signaling and E-cadherin. Canonical Wnt signaling activates *DPAGT1* expression and protein *N*-glycosylation, leading to extensive *N*-glycosylation of E-cadherin and weak intercellular adhesion. In OSCC, this positive feedback loop between Wnt signaling and *DPAGT1* is amplified, in part, by diminished expression of DKK-1, a canonical Wnt inhibitor. Furthermore, extensive *N*-glycosylation of E-cadherin prevents it from depleting nuclear β- and γ-catenins, allowing the positive feedback between Wnt and *DPAGT1* to operate without controls. (**B**) CTHRC1 is induced by *DPAGT1* and canonical Wnt signaling in OSCC. Schematic showing the interactions between *DPAGT1* and canonical Wnt signaling and their subsequent effects on CTHRC1 expression and non-canonical Wnt signaling.

Direct crosstalk between E-cadherin adhesion, *DPAGT1* and canonical Wnt signaling is demonstrated by studies showing that upregulation of *DPAGT1* by canonical Wnt leads to increased modification of E-cadherin with complex *N*-glycans, shown to be inhibitory to adhesion (Figure 4A) (Sengupta et al. 2010). Conversely, canonical Wnt activity and *DPAGT1* expression are downregulated with cell density and maturation of AJs (Jamal et al. 2012; Sengupta et al. 2013). Indeed, transfection of the hypo-glycosylated V13 E-cadherin mutant, but not fully N-glycosylated wild-type E-cadherin, into sparse MDCK or A253 cells, depletes nuclear β- and γ-catenin levels, consequently inhibiting canonical Wnt signaling and *DPAGT1* expression (Jamal et al. 2012; Sengupta et al. 2013). Both in dense cultures and in V13-transfected cells, reduction of nuclear γ-catenin is associated with its preferential recruitment to AJs, whereas depletion of nuclear β-catenin occurs via mechanisms that attenuate its cellular abundance. Therefore, mature AJs inhibit *DPAGT1* expression and canonical Wnt signaling by depleting nuclear β- and γ-catenins from the *DPAGT1* promoter (Figure 4A).

E-cadherin antagonizes canonical Wnt signaling and inhibits cell proliferation (Figure 4A) (Gottardi et al. 2001; Stockinger et al. 2001; Kam and Quaranta 2009; Maher et al. 2009). Inappropriate dissociation of β-catenin from E-cadherin leads to loss of adhesion, driving cancer development and progression. In human OSCC specimens, *DPAGT1* expression and canonical Wnt signaling are aberrantly induced, leading to extensive N-glycosylation of E-cadherin with complex *N*-glycans and loss of intercellular adhesion. Such extensive N-glycosylation of E-cadherin compromises its ability to inhibit canonical Wnt signaling and *DPAGT1* expression. Partial inhibition of *DPAGT1* with siRNA enhances E-cadherin adhesion and inhibits canonical Wnt signaling in OSCC cells, promoting changes in cellular morphology resembling a mesenchymal–epithelial transition (Nita-Lazar et al. 2009; Jamal et al. 2012). This is coincident with enhanced E-cadherin adhesion through the recruitment of stabilizing proteins to AJs. Likewise, transfection of OSCC cells with hypo-glycosylated E-cadherin inhibits *DPAGT1* expression and canonical Wnt signaling by depleting nuclear β- and γ-catenins from the *DPAGT1* promoter and from Wnt target genes (Jamal et al. 2012). Therefore, it is tempting to speculate that partial inhibition of *DPAGT1* may be an effective way to restore normal interactions among these pathways in OSCC.

### Dysregulation of DPAGT1/Wnt signaling in oral cancer: CTHRC1 and the non-canonical Wnt/PCP pathway

Dysregulation of the *DPAGT1*/canonical Wnt feedback loop has deleterious consequences to cellular homeostasis. Recent work demonstrates that in OSCC, *DPAGT1* and canonical Wnt signaling converge to induce the expression of CTHRC1, an *N*-glycoprotein implicated in invasion and metastasis of many aggressive tumors (Tang et al. 2006; Wang et al. 2012; Liu et al. 2013; Park et al. 2013). In human OSCC specimens, amplification of CTHRC1 levels is associated with its hyper-glycosylation. Partial inhibition of *DPAGT1* expression in OSCC CAL27 cells reduces CTHRC1 abundance by increasing protein turnover, indicating that N-glycosylation promotes CTHRC1 stability. Additionally, in OSCC, amplified canonical Wnt signaling induces β-catenin nuclear activity at the *CTHRC1* promoter to further increase CTHRC1 abundance (Liu et al. 2013) (Figure 4B).

In pancreatic cancer and OSCC CAL27 cells, CTHRC1 drives cell migration (Liu et al. 2013; Park et al. 2013). Recent studies suggest that *DPAGT1*-dependent induction of CTHRC1 facilitates its localization to cells at the leading edge of a wound front to promote OSCC cell migration (Liu et al. 2013). Although the precise mechanism by which CTHRC1 drives cell migration is unclear, CTHRC1 is implicated in the activation of the Wnt/planar cell polarity (PCP) pathway—a non-canonical branch of the Wnt pathway (Yamamoto et al. 2008). This relationship with the PCP pathway may also be relevant in OSCC, as CTHRC1 interacts with components of the non-canonical Wnt/PCP signaling including the Wnt5a ligand and the Fzd6 receptor (Liu et al. 2013). Accordingly, CAL27 cells and OSCC specimens display increased levels of non-canonical Wnt/PCP pathway components, including Dishevelled2 (DVL2), RAC1, RHOA and active JNK, p-JNK. Thus, aberrantly activated canonical Wnt signaling and N-glycosylation in OSCC collaborate to induce CTHRC1 on transcriptional and posttranslational levels, respectively, to drive OSCC cell migration and tumor spread (Figure 4B).

### N-Glycosylation and other signaling pathways in oral cancer

Canonical Wnt signaling and N-glycosylation affect a multitude of genes and proteins that function within diverse networks of signaling cascades that regulate cell proliferation, survival and motility. Many of these signaling pathways are known to have important roles in carcinogenesis, although the precise mechanisms linking them to canonical Wnt and N-glycosylation in specific cancers await further studies. Nonetheless, several signaling pathways that are dysregulated in OSCC are likely to be affected by the *DPAGT1*/canonical Wnt signaling feedback loop.

Major advances in the understanding how *N*-glycans impact cell behavior in cancer have come from studies revealing the association between high multiplicity of N-glycosylation sites and proliferation-promoting activities of receptor tyrosine kinases (RTKs) (Partridge et al. 2004; Lau et al. 2007; Lau and Dennis 2008; Dennis et al. 2009). RTKs are cell surface receptors for many growth factors, cytokines and hormones, and act to regulate a wide range of critical signaling pathways. At the cell surface, RTKs are regulated by a galectin lattice, which is a molecular structure formed between the Golgi-remodeled complex *N*-glycans and *N*-acetyl lactosamine-binding proteins, galectins. The Golgi enzymes have been shown to be ultrasensitive to UDP-GlcNAc flux for the formation of tri- and tetra-antennary *N*-glycans on extracellular domains of RTKs that preferentially interact with galectins. The galectin lattice has been shown to play key roles in preventing RTK diffusion and opposing the loss to endocytosis (Partridge et al. 2004; Lau et al. 2007). These important studies have established a conceptual framework for the current understanding of the role of N-glycosylation in RTK signaling in homeostasis and cancer.

Several RTKs are commonly dysregulated in OSCC, including EGFR and FGFR (Haugsten et al. 2010; Wheeler et al. 2010; Stransky et al. 2011; Motahhary et al. 2012). EGFR is aberrantly activated in ∼80% of head and neck cancers, frequently through unwarranted increases in its protein levels (Cassell and Grandis 2010; Wheeler et al. 2010). Elegant studies have shown that EGFR and FGFR belong to RTKs with high multiplicity of N-glycosylation sites (8–12), which has been aligned with their proliferation-promoting and oncogenic activities (Lau et al. 2007; Dennis et al. 2009). The Golgi-modified *N*-glycans regulate EGFR activity by controlling its surface retention within the galectin lattice and by preventing its loss to endocytosis. In cancer, aberrant upregulation of *MGAT5*, which promotes modification of *N*-glycans with poly-*N*-acetyllactosamine, stabilizes EGFR at the cell surface and promotes its activity (Lajoie et al. 2007; Dennis et al. 2009). Since upregulation of *DPAGT1* expression in OSCC is associated with increased modification of glycoproteins with complex *N*-glycans (Liu et al. 2013), it is likely that in the absence of mutations these structures are responsible for the observed increased activities of EGFR and FGFR in oral cancer. Further, EGFR expression may be regulated on a transcriptional level by β-catenin and the canonical Wnt pathway (Guturi et al. 2012). EGFR, in turn, activates downstream signaling cascades, including STAT, JNK and PI3K/AKT, known to promote tumorigenesis in oral cancer (Wheeler et al. 2010; Lui et al. 2013). Inhibition of N-glycosylation with tunicamycin disrupts RTK signaling in tumor cells and enhances susceptibility of lung cancer cells to a therapeutic agent, erlotinib, emphasizing the importance of *DPAGT1*/N-glycosylation in RTK signaling in cancer (Contessa et al. 2008; Ling et al. 2009). EGFR itself regulates β-catenin and Wnt signaling (Lee et al. 2010) and associates with E-cadherin, although the precise role of this interaction in OSCC has not been examined.

Type II transforming growth factor-beta (TGFβ) receptors, which bind to TGFβ and form complexes with Type I receptors to promote intracellular signals, are modified by *N*-glycans. In contrast to EGFR and FGFR, which display hyperbolic responses in cell surface expression in response to UDP-GlcNAc concentration, TGFβ receptors have a low number of *N*-glycan sites and their surface expression follows a sigmoidal response (Lau et al. 2007). Under normal conditions, this regulation assures that initial activation of proliferation by high multiplicity RTKs will be opposed by an increased presentation of TGFβ receptors at the cell surface, where they function to inhibit cell proliferation. However, this balance is lost in tumorigenesis when membrane remodeling and changes in cellular metabolism promote retention and activities of EGFR and other high multiplicity RTKs at the expense of TGFβ receptors. In addition, TGFβ family members are modified by *N*-glycans, and evidence suggests that N-glycosylation controls their stability, thereby shaping TGFβ morphogen gradients (Le Good et al. 2005). By controlling the transport and surface exposure of the TGFβ receptors, N-glycosylation may enable inappropriate binding to TGFβ (Kim et al. 2012). Dysregulated TGFβ signaling promotes disease progression, and thus aberrant N-glycosylation signaling may be a key mechanism facilitating TGFβ-induced oncogenic signals (Massague 2008), including those in OSCC (Prime et al. 2004).

Tumor cells are frequently hyper-metabolic (a phenomenon known as the Warburg effect) due to their preferred production of energy by aerobic glycolysis rather than oxidative phosphorylation (Ward and Thompson 2012). Indeed, glucose has been shown to be required for the proliferation and survival of head and neck squamous carcinoma cells (Sandulache et al. 2011). High rates of glycolysis are likely to augment the levels of UDP-GlcNAc, a substrate for GPT, derived from the hexosamine pathway (Anagnostou and Shepherd 2008; Dennis et al. 2009). Accoringly, the roles of metabolism and UDP-GlcNAc in the regulation of the Golgi N-glycosylation in homeostasis and cancer have been described (Mendelsohn et al. 2007; Dennis et al. 2009). Furthermore, increased glucose levels also induce canonical Wnt signaling and protein N-glycosylation in cultured cells, and this has been aligned with more extensive N-glycosylation of EGFR (Konishi and Berk 2003; Anagnostou and Shepherd 2008). The *DPAGT1* promoter has potential binding sites for HIF1, a transcription factor known to influence the metabolic shift during tumorigenesis (Figure 2). HIF1 is stabilized under hypoxia and triggers the upregulation of genes that drive the switch to glycolysis, including GLUT1, HKII and PDK1 (Harada et al. 2009). Furthermore, p53, a tumor suppressor most frequently inactivated in oral cancer (Agrawal et al. 2011; Sano et al. 2011), also has potential binding sites in the promoter of *DPAGT1* (Figure 2). Since glycolytic enzymes are inhibited by pharmacologically activated p53, it is possible that under normal conditions of homeostasis, p53 acts as a suppressor of unwarranted N-glycosylation (Zawacka-Pankau et al. 2011). Thus, tumor cells may upregulate *DPAGT1* expression by increasing substrate levels and/or by increasing transcriptional regulation as a consequence of activated oncogenic factors, such as HIF1. Further studies, however, are required to examine these ideas.

## Conclusions

N-Glycosylation, canonical Wnt signaling and E-cadherin adhesion belong to a core network of cellular processes that maintain a fine balance between proliferation and adhesion. These processes play pivotal roles in embryogenesis and their dysregulation is a feature of many malignancies, including OSCC. How this network becomes dysregulated in cancers is still poorly understood. It is possible that the initial activation of the *DPAGT1*/canonical Wnt feedback loop may involve a mutation in any of the components of the canonical Wnt signaling pathway. Likewise, upregulation of *DPAGT1* expression either by activating mutations or by increased availability of UDP-GlcNAc from glycolysis may initiate the deregulation of the network. Since activation of canonical Wnt signaling and protein N-glycosylation contributes to EMT, it is also likely that this N-glycosylation/Wnt network plays a role in metastasis (Fodde and Brabletz 2007; Dennis et al. 2009; Polyak and Weinberg 2009). EMT underlies many aggressive features of head and neck cancers and is associated with the acquisition of stem cell-like properties in cancer, including OSCC (Prince et al. 2007; Mandal et al. 2008; Mani et al. 2008). Interestingly, canonical Wnt signaling is required for the maintenance of stem cell niches in various tissues, including skin, intestinal crypt, hair follicles and mammary gland (Korinek et al. 1998; Wend et al. 2010; Schwitalla et al. 2013). Most recently, induced canonical Wnt signaling has been shown to be a feature of cancer initiating cells in human salivary gland and head and neck tumors (Wend et al. 2013). Thus, it is tempting to speculate that dysregulation of *DPAGT1*/canonical Wnt feedback loop may have an important role in directing “stem cell-like” properties in cancers.

We propose that inappropriate activation of either *DPAGT1* or canonical Wnt signaling sets off a sequence of events that lead to hyper-glycosylation and transcriptional activation of oncogenic transcriptional targets, such as CTHRC1 and EGFR. These events subsequently have the potential to drive feedforward pro-tumorigenic signals. Future studies are likely to reveal the molecular details into how these signals are impacted by the N-glycosylation/Wnt interplay and whether distinct N-glycosylation-regulated events control different stages of tumorigenesis. Given the large increases of N-glycosylation in cancers, along with the impact that dysregulated N-glycosylation can have on a range of signaling processes, targeting *DPAGT1* or other N-glycosylation pathway regulators represents a potential approach for cancer treatment.

## Funding

M.A.K. was supported by the National Institutes of Health (RO1 DE015304 and RO1 DE014437) and X.V. by the March of Dimes Foundation (Research Grant number 5-FY11-578) and the Concern Cancer Foundation. Funding to pay the Open Access publication charges for this article was provided by the Boston University School of Dental Medicine Oral Cancer Research Initiative to M.A.K.

## Conflict of interest statement

None declared.

## Abbreviations

AJs, adherens junctions; CHO, Chinese Hamster Ovary; CTHRC1, collagen triple helix repeat containing 1; DRDs, dolichol recognition domains; DVD2, Dishevelled2; ECs, ectodomains; EMT, epithelial-to-mesenchymal transition; ER, endoplasmic reticulum; GlcNAc, *N*-acetylglucosamine; GPT, dolichol phosphate-dependent *N*-acetylglucosamine 1-phospho-transferase; LLO, lipid-linked oligosaccharide; MDCK, Madin-Darby canine kidney; N-cadherin, neural cadherin; OSCC, oral squamous cell carcinoma; PCP, planar cell polarity; PP2A, protein phosphatase 2A; RTKs, receptor tyrosine kinases; TGFβ, transforming growth factor-beta; TJs, tight junctions.
